# Novel Diabetic Mouse Models as Tools for Investigating Diabetic Retinopathy

**DOI:** 10.1371/journal.pone.0049422

**Published:** 2012-12-12

**Authors:** Peter F. Kador, Peng Zhang, Jun Makita, Zifeng Zhang, Changmei Guo, James Randazzo, Hiroyoshi Kawada, Neena Haider, Karen Blessing

**Affiliations:** 1 Department of Pharmaceutical Sciences, College of Pharmacy, University of Nebraska Medical Center, Omaha, Nebraska, United States of America; 2 Department of Ophthalmology, College of Medicine, University of Nebraska Medical Center, Omaha, Nebraska, United States of America; 3 Department of Genetics, Cell Biology and Anatomy, College of Medicine, University of Nebraska Medical Center, Omaha, Nebraska, United States of America; National Eye Institute, United States of America

## Abstract

**Objective:**

Mouse models possessing green fluorescent protein (GFP) and/or human aldose reductase (hAR) in vascular tissues have been established and crossed with naturally diabetic Akita mice to produce new diabetic mouse models.

**Research Design and Methods:**

Colonies of transgenic C57BL mice expressing GFP (SMAA-GFP), hAR (SMAA-hAR) or both (SMAA-GFP-hAR) in vascular tissues expressing smooth muscle actin were established and crossbred with C57BL/6-Ins2^Akita^/J (AK) mice to produce naturally diabetic offspring AK-SMAA-GFP and AK-SMAA-GFP-hAR. Aldose reductase inhibitor AL1576 (ARI) was administered in chow. Retinal and lenticular sorbitol levels were determined by HPLC. Retinal functions were evaluated by electroretinography (ERGs). Growth factor and signaling changes were determined by Western Blots using commercially available antibodies. Retinal vasculatures were isolated from the neural retina by enzymatic digestion. Flat mounts were stained with PAS-hematoxylin and analyzed.

**Results:**

Akita transgenics developed DM by 8 weeks of age with blood glucose levels higher in males than females. Sorbitol levels were higher in neural retinas of AK-SMAA-GFP-hAR compared to AK-SMAA-GFP mice. AK-SMAA-GFP-hAR mice also had higher VEGF levels and reduced ERG scotopic b-wave function, both of which were normalized by AL1576. AK-SMAA-GFP-hAR mice showed induction of the retinal growth factors bFGF, IGF-1, and TGFβ, as well as signaling changes in P-Akt, P-SAPK/JNK and P-44/42 MAPK that were also reduced by ARI treatment. Quantitative analysis of flat mounts in 18 week AK-SMAA-GFP-hAR mice revealed increased loss of nuclei/capillary length and a significant increase in the percentage of acellular capillaries present which was not seen in AK-SMAA-GFP-hAR treated with ARI.

**Conclusions/Significance:**

These new mouse models of early onset diabetes may be valuable tools for assessing both the role of hyperglycemia and AR in the development of retinal lesions associated with diabetic retinopathy.

## Introduction

Diabetic Retinopathy (DR) is primarily a microvascular complication where a selective degeneration of retinal capillary pericytes occurs. This results in a retinal capillary pericyte to endothelial cell ratio increase from 1∶1 in nondiabetics to up to 1∶18 in diabetic patients [1], [2]. Pericytes regulate capillary tone, control retinal capillary blood flow through their contractile nature (presence of smooth muscle actin) and control neovasculogenesis by suppressing the growth of endothelial cells [3], [4], [5].

Animal studies have played a critical role in elucidating the mechanism(s) of how retinal lesions are induced by hyperglycemia. Studies have also established that similar hyperglycemic-associated retinal lesions also develop in galactose-fed animals; however, the retinal lesions develop faster and are more severe in galactosemic animals. For example, while retinal lesions in diabetic dogs generally do not develop past background retinopathy, galactose-fed dogs, develop retinal changes that include the proliferative stage [6], [7], [8], [9], [10], [11], [12]. In fact, the galactose-fed dog is the first animal model to clearly support the hypothesis that pericyte loss is the hallmark of DR proposed by Cogan and Kuwabara. Pericyte loss is followed by either an adjacent increased focal growth of endothelial cells to form microaneurysms, or the subsequent degeneration of endothelial cells to form acellular capillaries through which blood, oxygen and nutrients no longer flow. Compared to pericyte loss, however, the loss of endothelial cells is not significant until the latter stages of retinopathy [8]. Further studies with canine retinal capillary cells indicate that aldose reductase (AR) is expressed in pericytes and that exposure of retinal capillary cells to high sugar levels initiates apoptosis in pericytes that is prevented by treatment with aldose reductase inhibitors (ARIs) [13], [14], [15].

The role of AR in retinal lesion formation has also been observed in diabetic and galactose fed rats [8], [10], [16], [17], [18]. However, in contrast to marked pericyte loss in dogs, rats demonstrate increased periodic-acid-Schiff (PAS) staining of retinal capillaries suggestive of basement membrane thickening that is prevented ARIs [19], [20]. While retinal capillary pericyte degeneration also occurs in both diabetic and galactose-fed rats, this degeneration is not as pronounced and occurs after significant capillary basement membrane thickening has developed. This pericyte degeneration is also prevented by ARIs [16], [21], [22]. *In vitro* culture of rat retinal capillary pericyte (TR-rPCT) and endothelial (TR-iBRB) cells in 50 mM glucose or galactose show significant polyol accumulation in pericytes compared to endothelial cells and results in increased TUNEL staining that was reduced by AR inhibition [23].

Compared to the dog or rat, mice represent a more versatile animal model for investigating the development of lesions associated with DR because select genes can easily be manipulated or removed. However, compared to rats, it is more difficult to induce diabetes in the mouse with alloxan or streptozotocin and to maintain the diabetic mouse for the prolonged time periods required to observe retinal lesions [24]. Distinguishing pericyte degeneration in mice is also more difficult because the similar appearance of pericyte and endothelial cell nuclei makes at least 25% of capillary cells in conventionally stained (PAS and hematoxylin) preparations difficult to distinguish [25], [26]. While several studies with transgenic and knock-out mice suggest that AR is also linked to the development of retinal lesions [17], [18], Obrosova has reported significant differences in retinal oxidative parameters between the rat and mouse retina and suggested that there are lower levels of AR in the mouse retina [27]. Here, we report the development of new transgenic mice expressing green fluorescent protein (GFP) and human aldose reductase (hAR) in all vascular cells containing smooth muscle actin (e.g. retinal capillary pericytes) that when cross bred with Akita mice develop diabetes. These diabetic transgenic mice expressing hAR demonstrate expressions changes in growth factor and signaling along with retinal capillary cell drop-out that are prevented by the administration of aldose reductase inhibitors.

## Materials and Methods

### Chemicals

All reagents and solvents were commercially obtained from Acros Organics and Fisher Scientific (Pittsburgh, PA) or Sigma-Aldrich Corporation (St Louis, Mo) and utilized without further purification. All solvents were reagent or HPLC grade. The ARI AL1576 (2,4-difluorospirofluorene-9,5′-imidazolidine-2′,4′-dione) was obtained from Alcon Laboratories (Ft. Worth, TX).

Antibodies utilized were as follows: GAPDH rabbit Ab, and mouse VEGF mAb (sc-147; VEGF-A) were obtained from Abcam Inc, Cambridge, MA. and IGF-1 rabbit mAb were obtained from Santa Cruz Biotech (Santa Cruz, CA); phospho-Akt (Ser473) rabbit Ab, phospho-ERK (phospho-44/42 MAPK) (Thr202/Tyr204) rabbit Ab, phospho-SAPK/JNK (Thr183/try185) rabbit mAb, TGF-β rabbit Ab, basic-FGF rabbit Ab, horseradish peroxidase (HRP) conjugated anti-rabbit antibody, and HRP conjugated anti-biotin Ab were obtained from Cell Signaling Technology (Beverly, MA). Chemiluminescent reagent and peroxide, biotinylated protein ladder, prestained protein marker, and cell lysis buffer were from Cell Signaling Technology (Beverly, MA).

### Animals

All studies were approved by the University of Nebraska Medical Center Institutional Animal Care and Use Committee and conformed to the Association for Research in Vision and Ophthalmology Statement for the Use of Animals in Ophthalmic and Vision Research.

### Mice Breeding

A breeding pair of mice transgenic for green fluorescent protein (GFP) and a pair transgenic for human aldose reductase (hAR) were obtained from the National Eye Institute, NIH. These mice were prepared by Dr. Jen-Yu Tsai while at the Laboratory of Ocular Therapeutics of the National Eye Institute while Kador was chief of the laboratory. The C57BL6-SMAA-GFP (GFP) mice that express GFP under the control of the smooth muscle alpha actin promoter and the C57BL6-SMAA-hAR (hAR) mice that express hAR under the control of smooth muscle alpha actin promoter were produced in the Transgenic Mice Facility of the National Eye Institute using the smooth muscle alpha actin promoter provided by Dr. James Fagin [28]. The regulatory sequence of the smooth muscle alpha actin gene used contains −1074 bp of the 5′ flanking region, the transcription start site, 48 bp of exon 1, the 2.5-kb intron 1, and the 15-bp exon 2 of mouse smooth muscle alpha actin. GFP is specifically expressed in both vascular and nonvascular smooth muscle cells [29], [30], [31], [32]. Similarly to GFP, hAR is expressed in both vascular and nonvascular smooth muscle cells; however, hAR was also found to be expressed in the cone bipolar cells [33]. The GFP mice were also cross bred with hAR mice to produce a colony of GFP-hAR mice.

Heterozygous C57BL/6-Ins2^Akita^/J (AK) mice were purchased from the Jackson Laboratory (Bar Harbor, MA). Female AK mice were initially mated to GFP males with 1 male and 2 females in each cage. Once pregnant, each female was individually housed. After birth, each GFP mouse in the litter was genotyped. Mice positive for both GFP and Ins2^Akita^ were retained. Similarly, female AK mice were mated with male GFP-hAR mice.

### Genotyping for SMAA-GFP

Genomic DNA was isolated from tail snips/ear punches by first digesting the tissue for a minimum of 5 hours at 56°C in Direct Lysis (Viagen Biotech) and Proteinase K solution (Invitrogen). The sample was then heated for 45 min. at 85°C. Polymerase chain reaction (PCR) analysis was conducted by mixing 1 µL of DNA sample with 33.5 µl of DD H_2_0, 10 µl of 5× Green Go Taq Flexi Buffer, 3 µL MgCl_2_ (25 mM), 1 µl of DNTP and 0.5 µl of each primer at 100 pM (SMAA-GFP-right: 5′- GAC GTA AAC GGC CAC AAG TTC AG -3′; SMAA-GFP-left: 5′- GAT GCG GTT CAC CAG GGT GTC G-3′, Integrated DNA technologies, Inc.). PCR was conducted according to the following conditions where the Lid temp is 104.5°C: a: 96°C for 4 min.; b: 96°C for 45 sec.; c: 55°C for 45 sec.; d: 72°C for 1 min (repeat b-d another 32 cycles = 33 total); e: 72°C for 5 min.; f: 4°C pause for storage. The product, 492 bp, was visualized by UV light following gel electrophoresis on a 2% Agarose gel containing ethidium bromide.

### Genotyping for SMAA-hAR

Tissues were obtained and prepared as described for genotyping for SMAA-GFP. Genotyping for SMAA-hAR was conducted by PCR using the primers SMAA-hAR-right: 5′-TGA GTG CCA CCC ATA TCT CA-3′ SMAA-hAR-left: 5′-AGG TGG CCA TAT CCT GGC TG-3′; (Integrated DNA technologies, Inc.). PCR was conducted according to the following conditions where the Lid temp was 104.5°C: a: 96°C for 4 min.; b: 96°C for 45 sec.; c: 55°C for 45 sec.; d: 72°C for 1 min (repeat b-d another 32 cycles = 33 total); e: 72°C for 5 min.; f: 4°C for pause for storage. The product, 311 bp, was be visualized by UV light following gel electrophoresis on a 2% Agarose gel containing ethidium bromide.

### Genotyping for Ins2^Akita^

Tissues were obtained and prepared as described for genotyping for SMAA-GFP. The PCR amplification of a −280 DNA fragment was amplified from both the wild type and mutant alleles using the primers (Ins2) IMR1093 primer 5′-TGC TGA TGC CCT GGC CTG CT -3′ together with 5′-TGG TCC CAC ATA TGC ACA TG -3′. PCR was conducted according to the following conditions where the Lid temp was 104.5°C: a: 96°C for 4 min; b: 96°C for 45 sec; c: 55°C for 45 sec (−0.5 C per cycle); d: 72°C for 60 sec (repeat steps b–d for 32 cycles = 33 total); 72°C for 5 min.; f: 4°C for pause for storage. A restriction enzyme was employed by mixing 1.2 µL of 10× NE Buffer 4 (New England Biolabs), 5.8 µL DD water and 1 µL Fnu_4_H restriction enzyme with 12 µL of the PCR product. The Fnu4H restriction enzyme is unable to digest the PCR products from the mutant allele so the product appears at 280 bp. Because the PCR products from the wild type allele can be digested by the Fnu4H restriction enzyme, the product appears at 140 bp. These bands were visualized following gel electrophoresis on a 2% Agarose gel containing ethidium bromide.

### ARI Administration

Select AK-SMAA-GFP-hAR mice were fed a standard rodent diet containing either 0.02% AL1576. Food consumption and body weights were monitored weekly. These records indicate that mice received an average dose of 11 mg/kg/day of AL1576 and 23 mg/kg/day of Ranirestat.

### Sugar Analysis

Three micromoles of xylitol was added as an internal standard to 4 lenses or 4 neural retinas in 1 mL of ice cold PBS and the mixture was homogenized. An aliquot of the homogenate was removed for colorimetric protein quantification using the DC Protein Assay (Bio-Rad Laboratories, Hercules, CA) compared against bovine serum albumin (BSA) protein standards. Each sample was then deproteinized by overnight centrifugation at 8°C through a Microcon YM-10 Centrifugal Filter Device and the filtrates were dried in a Speedvac. Each dried residue was dissolved in 900 µL of pyridine and then derivatized with 900 µL of phenyl isocyanate at 55°C for 60 min. After cooling the samples in an ice bath, the reaction was halted with cold methanol. This was again followed by heating for 5 min. The derivatized samples were analyzed by HPLC on an automated Hewlet Packard 1100 Chemstation equipped with a diode array detector. Samples (5 µl) were injected onto a 150×4.6 mm Tosoh TSK-GEL ODS-80Tm column containing a 3.2×15 mm guard column at 35°C. Samples were eluted isocratically with 20 mM potassium phosphate/acetonitrile (35∶65 v%), pH 7.0, at a flow rate of 1.0 ml/min and detected at 235 nm. Samples were quantified against standard curves of glucose, galactose, sorbitol, galactitol, myo-inositol, xylose (0.008–6.0 µmol).

### SDS-PAGE and Western Immunoblot Analyses

Neural retinas were carefully removed from the two posterior segment of each mouse and the combined retinas were immediately frozen over dry ice. The thawed retinas were sonicated with ice cold lysis buffer (1% NP-40, 0.5% Deoxycholate, 1% SDS, 150 mmol/L NaCl, 50 mmol/L Tris–HCl, pH = 8) supplemented with a mixture of protease inhibitors (Cell Signaling Technology, USA). In soluble protein in each retinal homogenate was removed by centrifugation in a microcentrifuge (14000 rpm, 30 min, 4°C). Protein levels in the remaining supernatant were measured according to the Bradford Assay [34]. Fifty micrograms of total protein from each homogenate was separated in precast linear 4–15% tris-HCl gradient polyacrylamide gel (Ready Gels, BIO-Rad, Hercules, CA). The separated proteins were electrophoretically transferred to nitrocellulose membrane, blocked with a 5% powdered milk solution and washed with tris-buffered saline (TBS). The membranes were then separately incubated overnight at 4°C with antibodies against bFGF, TGF-β, IGF-1, P-Akt, P-ERK1/2, and P-SAPK/JNK in accordance with the manufacturer's instructions. After final washings with 0.05% TBS-Tween, membranes bound antibody complexes were visualized by applying HRP conjugated anti-rabbit antibody to the membrane for 1 hr at room temperature. The blots were again washed with TBS and processed for chemiluminescence detection of the immunoreactive proteins after incubation for 5 min at room temperature. Immunoreactive band densities were measured using Image-Pro Plus software (Bethesda, MD) and the NIH ImageJ image analysis program (1.42q).

### Electroretinography (ERG)

ERG studies were conducted on a minimum of 3 SMAA-GFP-hAR mice, AK-SMAA-GFP-hAR mice, and AK-SMAA-GFP-hAR mice treated with-AL1576. Each mouse was prepared for ERG analysis under dim red light illumination. Pupils were first dilated with 1% atropine sulfate. The mice were then anesthetized with an i.p. injection of ketamine (100 mg/kg) and xylazine (20 mg/kg). A drop of 1% carboxymethylcellulose was placed on the corneal surface of each eye to ensure electrical contact and to maintain corneal integrity. This was followed by the placement of the ERG electrodes (LKC Technologies Inc., Gaithersburg, MD). A needle electrode, placed under the skin of the forehead, served as ground. Body temperature was maintained at 38±0.3°C using a Themipaq heating pad. ERG recordings were performed according to Nystuen [35] using the UTAS system (LKC Technologies Inc., Gaithersburg, MD). Briefly, all stimuli generated were presented in a Ganzfeld chamber/light emitter. Dark-adapted (scotopic) responses were recorded over a 4.0 log unit range of intensities. Light-adapted (photopic) responses were obtained with white flashes (0.3 log unit step) after 10 min of exposure to the background light to allow for complete light adaptation. Flicker ERG analysis was not performed. The amplitude of the scotopic a-wave was measured from baseline to the a-wave trough, while the amplitude of the scotopic b-wave was measured from the trough of the a-wave to the peak of the b-wave. The photopic b-wave amplitude was measured from baseline to the b-wave peak.

### Preparation of Retinal Digests

Enucleated eyes were cut at the limbus and fixed at room temperature in a solution of 4.0% w/v paraformaldehyde in 50 mmol/l sodium/potassium phosphate buffer with 6.0% sucrose at pH 7.2. After a minimum of 4 days of fixation, the anterior segment was removed by careful microdissection at the ora serrata and the intact fixed neural retina was carefully removed from the posterior globe. The fixed retina was washed with deionized water for 4–6 hr and then with gentle agitation incubated at 37°C with 100 mmol/l sodium phosphate buffer, pH 6.5, containing 40 units/ml elastase, 150 mmol/l sodium chloride and 5.0 mmol/l ethylenediamene tetraacetic acid (EDTA) (elastase solution). After 15–20 min. the partially digested retina was washed overnight at room temperature with 100 mmol/l Tris-HCl buffer pH 8.5. The retinas were then transferred to deionized water and the loosened vitreous and digested neural elements were gently removed from the retinal vasculature by agitation. Tissue adhering to the retinal vasculature after this step was removed by additional 3–5 min. incubation in fresh elastase solution for 3–5 min. followed by a second overnight wash in fresh Tris-HCl buffer and then gentle agitation in deionized water. All remnants of optic nerve were removed and peripheral cuts were made into the cup-shaped isolated retinal vasculature bed as needed to permit an even flattening of the preparation. The vascular preparation was then mounted by flotation with calcium-magnesium-free Dulbecco's phosphate-buffered saline over slides coated with 0.25% gelatin. Following air drying in a dust free environment, the adhering retinal vasculature preparations were stained with periodic acid Schiff (PAS) and then hematoxylin.

### Analysis of Retinal Digests

Each retinal preparation was divided into 4 equal quadrants and the central areas in each quadrant mid-distance between the optic nerve and outer retinal edge were analyzed. At the central point in each of these quadrants, 4 adjacent 230×300 micron areas were captured with an Olympus BX51 research microscope at 400× magnification and analyzed using PAX-it Image software (Chicago, IL). Approximately 1.1 mm^2^ central areas of each mouse retina were analyzed. Retinas from a minimum of four 18 week diabetic AK-SMAA-GFP-hAR and AK-SMAA-GFP-hAR mice treated with AL1576 along with age-matched nondiabetic SMAA-GFP-hAR mice were analyzed.

### Statistical Analyses

The calculations and statistical analyses (ANOVA and 2-sample t-test were conducted using OriginPro® software version 8.1 (OriginLab Corp., Northampton, MA) and ProStat ver. 5.01 (Pearl River, NY). Differences with a p<0.05 were defined as significant.

## Results

A colony of transgenic mice (SMAA-GFP) where green fluorescent protein (GFP) was introduced in all vascular tissues containing smooth muscle actin to aid in the identification of retinal capillary pericytes was established from a breeding pair of transgenic C57BL6-SMAA-GFP mice expressing GFP under the control of smooth muscle alpha actin promoter. Similarly, a colony of transgenic mice (SMAA-hAR) possessing human aldose reductase (hAR) in all vascular tissues containing smooth muscle alpha actin was established from a breeding pair of C57BL6-SMAA-hAR mice expressing hAR under the control of smooth muscle alpha actin promoter. Inbreeding within each respective colony resulted in approximately 100% of all offspring demonstrating the presence of GFP or hAR. However, mating efficiency drastically decreased when essentially all offspring displayed the desired traits. Optimal breeding efficacy in both colonies required the intermittent addition of wild type C57BL6 mice. In the SMAA-GFP colony, optimal breeding efficiency was observed when approximately 70–75% of offspring expressed GFP, while in the SMAA-hAR colony optimal breeding efficiency was observed when approximately 40–45% of offspring expressed hAR. The SMAA-GFP and SMAA-hAR mice were also crossbred to establish an additional colony of mice containing both GFP and hAR traits. With repeated inbreeding both traits were present in >95% of all offspring. Traits in all offspring were determined by genotyping DNA isolated from tail snips/ear punches with PCR.

**Figure 1:**
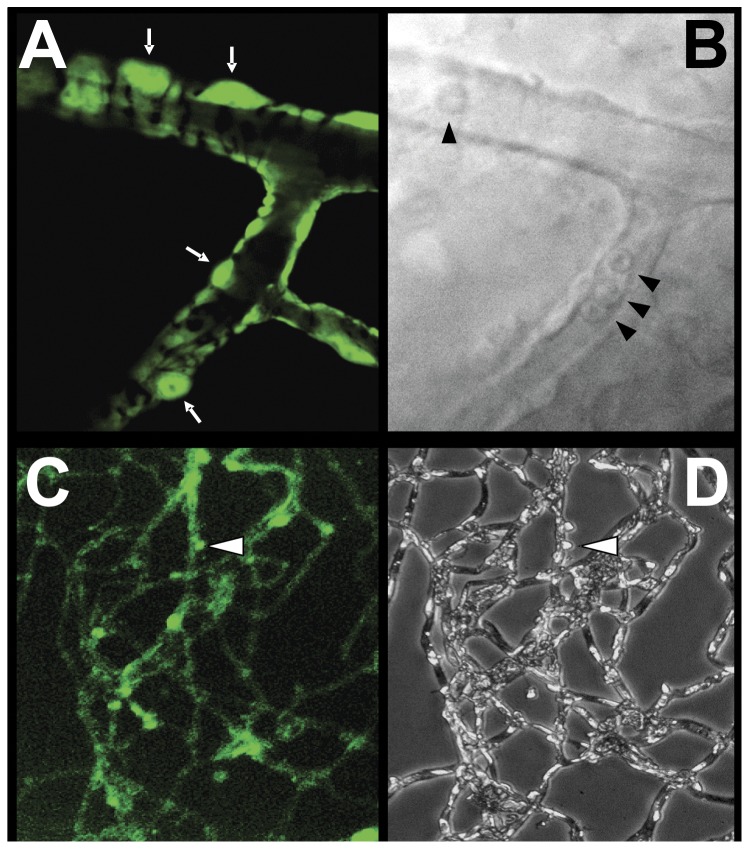
Appearance of retinal capillaries from transgenic mice. (**A**) Confocal microscopy of a retinal capillary from an SMAA-GFP mouse illustrating the appearance of pericytes with their finger-like cytoplasmic projections compassing the vascular endothelial cells. GFP is present in the cytoplasm of the pericytes and their processes (white arrows). (**B**) Bright field image of the same capillary showing the presence of red blood cells (arrowheads). (**C**) Appearance of retinal capillaries isolated by trypsin digestion illustrating that GFP is retained in the intact pericytes and can be used for their identification. (**D**) Appearance of the same capillaries by light microscopy.

In the SMAA-GFP mice, the tissue presence of GFP can easily be detected by fluorescent microscopy. This includes not only the retina but also vascular tissues in the brain, kidney, heart, lung and mammary glands. Confocal microscopy of the retinal capillaries confirmed that GFP is present in the finger-like cytoplasmic projections of pericytes that encompass the vascular endothelial cells but not in the endothelial cells themselves (Fig. 1). GFP is also retained within the cytoplasm of the pericytes when the retinal capillaries are carefully isolated by trypsin digestion (Fig. 1). This confirms that the selective presence of GFP in the pericytes may be a useful tool for differentiating pericytes from endothelial cells in isolated retinal capillaries.

Diabetes was introduced into these colonies of transgenic mice by breeding SMAA-GFP and SMAA-GFP-hAR expressing mice with naturally diabetic C57BL/6-Ins^2Akita^/J (AK) mice which carry a dominant mutation in the Mody4 locus on chromosome 7 in the insulin 2 gene [36]. Better mating was obtained when diabetic AK females were mated with SMAA-GFP-hAR or SMAA-GFP males to produce AK-SMAA-GFP-hAR or AK-SMAA-GFP offspring. This may be due to the fact that AK females are less hyperglycemic than males and actively breed longer. When SMAA-GFP-hAR and AK mice were mated, approximately 27% of offspring (males>females) became diabetic by 8 weeks of age. Breeding either two AK-SMAA-GFP-hAR or two AK-SMAA-GFP mice together did not produce a higher rate of heterozygous diabetic mice.

**Figure 2:**
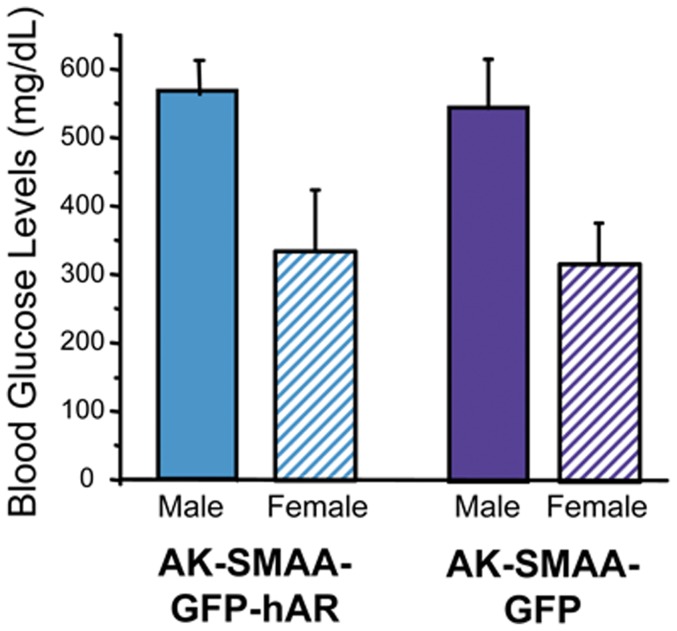
Comparison of blood glucose levels in male and female diabetic AK-SMAA-GFP and AK-SMAA-GFP-hAR mice. Similar blood glucose levels were observed with both groups of mice with levels higher in males than females. n = 4–6; mean ± S.D.

**Figure 3:**
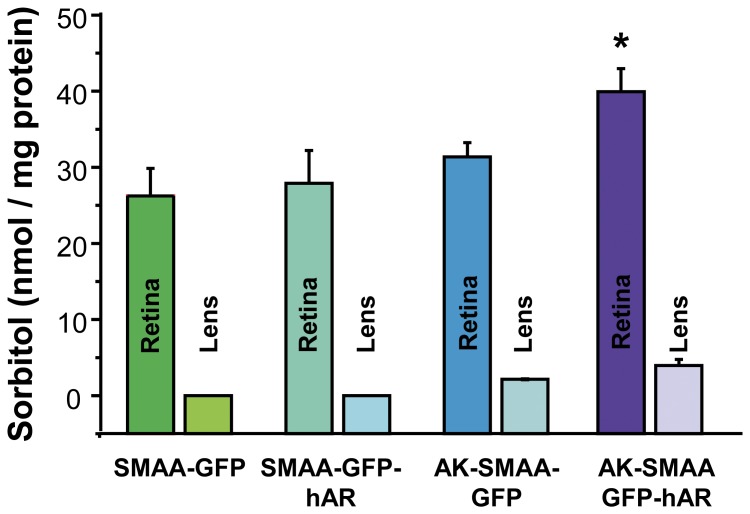
Comparison of sorbitol levels in the isolated neural retinas of non-diabetic SMAA-GFP and SMAA-GFP-hAR mice and AK-SMAA-GFP and AK-SMAA-GFP-hAR mice. Significantly increased sorbitol levels were observed in the AK-SMAA-GFP-hAR mice. n = 4–6; mean ± S.E.M. * p<0.05.

In the AK crossed mice, diabetes develops by 8 weeks with blood sugar levels similar in both the AK-SMAA-GFP-hAR and the AK-SMAA-GFP mice; however, males have significantly higher blood sugar levels than females (Fig. 2). Sorbitol levels in the neural retinas from AK-SMAA-GFP-hAR mice are significantly higher (25%) than those from AK-SMAA-GFP mice. This is attributed to the increased presence of hAR in the neural retina. The sorbitol levels in the neural retinas from diabetic AK-SMAA-GFP mice are not significantly higher than the sorbitol levels in the neural retinas from nondiabetic SMAA-GFP or SMAA-GFP-hAR mice (Fig. 3). For the present studies, the average blood sugar levels (mean ± SEM) for the AK-SMAA-GFP mice was 481±27 mg/dL, for the AK-SMAA-GFP-hAR mice, 502±20 mg/dL, and for the AK-SMAA-GFP-hAR mice treated with AL1576, 499±19 mg/dL.

**Figure 4:**
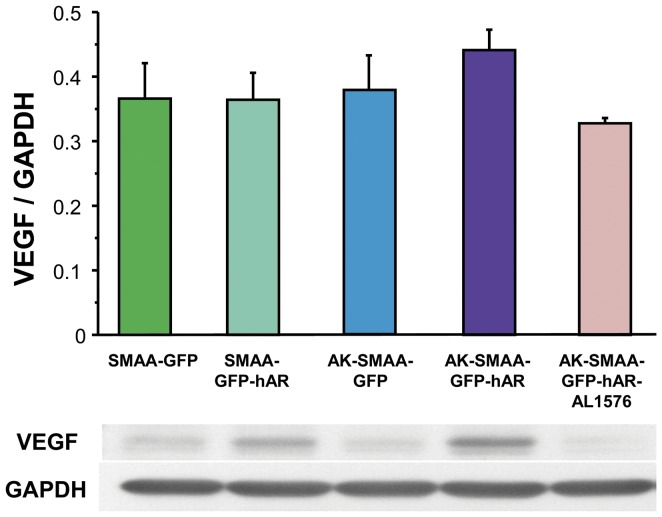
Induction of VEGF in neural retinas from 18 week old non-diabetic SMAA-GFP and SMAA-GFP-hAR mice and AK-SMAA-GFP and AK-SMAA-GFP-hAR mice. Highest VEGF induction was observed in AK-SMAA-GFP-hAR mice which was reduced in similar mice treated with the ARI AL1576. Bars represent the mean ± S.E.M of a minimum of 3 separate analyses. The gel illustrates the representative appearance of one analysis.

**Figure 5:**
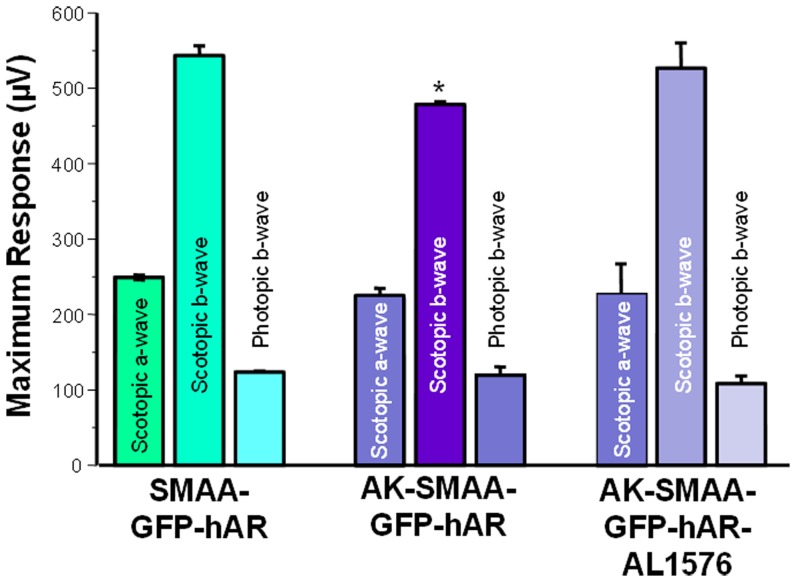
Comparison of ERG maximum scotopic b waves obtained from nondiabetic SMAA-GFP-hAR and AK-SMAA-GFP-hAR mice. n = 3–4; mean ± S.E.M. * p<0.05.

In diabetic rats, elevated retinal sorbitol levels have been linked to increased VEGF expression and decreased b-wave electroretinogram (ERG) pattern responses. Both changes are normalized by treatment with ARIs [37], [38], [39], [40], [41]. Comparison of VEGF expression levels in the neural retinas from 18 week old SMAA-GFP, SMAA-GFP-hAR, AK-SMAA-GFP and AK-SMAA-GFP-hAR mice showed that the expression levels of VEGF-A only increased in the neural retinas from AK-GFP-hAR mice and this expression was reduced when AK-SMAA-GFP-hAR mice were treated with the ARI AL1576 (Fig. 4). ERG pattern changes in SMAA-GFP-hAR mice were also compared to those in AK-SMAA-GFP-hAR mice treated with/without the AL1576. A significant decrease in the scotopic but not photopic b-wave, was observed in AK-SMAA-GFP-hAR mice (Fig. 5). This decrease was normalized in similar AK-SMAA-GFP-hAR mice treated with AL1576.

**Figure 6:**
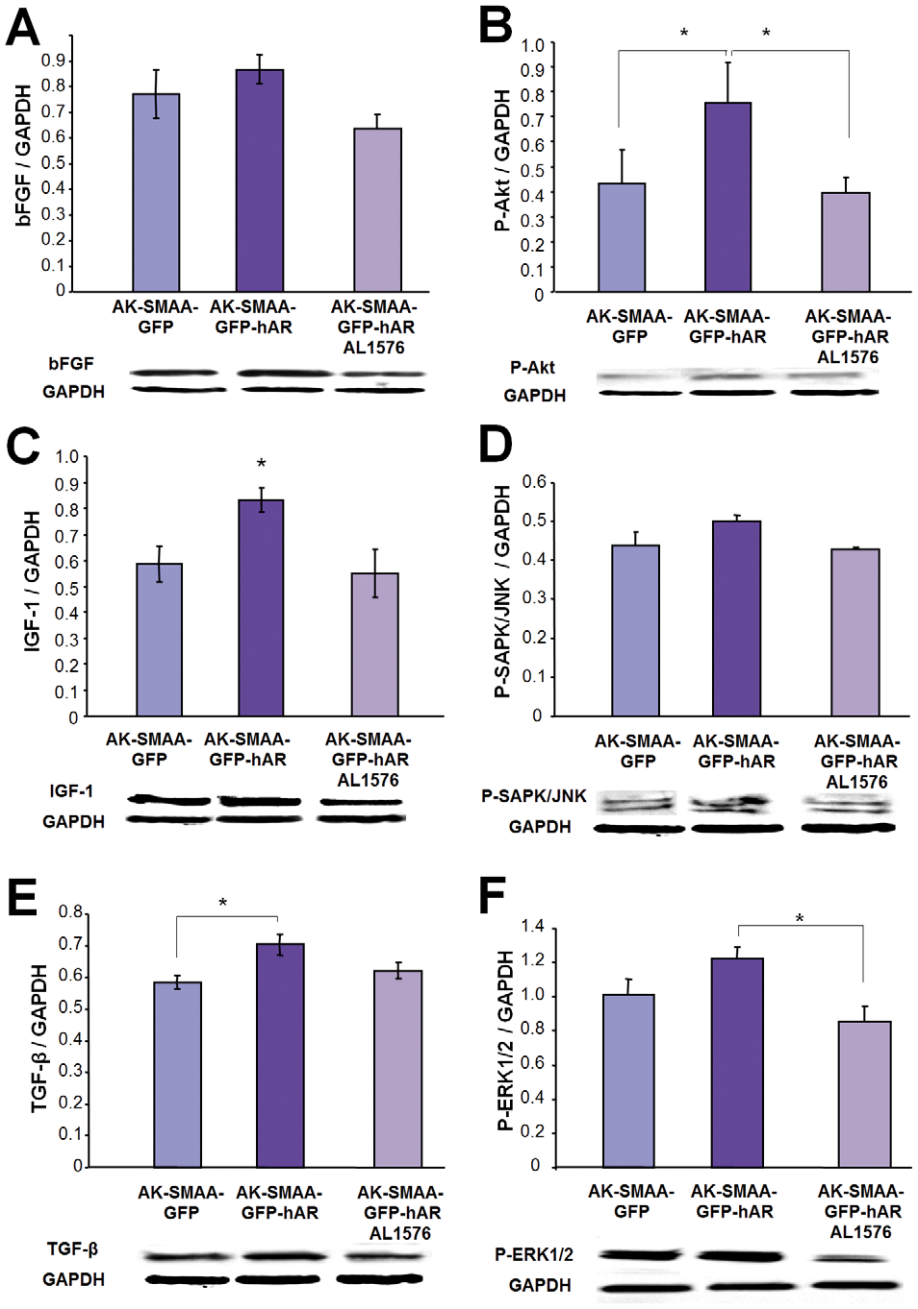
Comparison of (A) bFGF, (C) IGF-1, and (E) TGF-β growth factor changes in AK-SMAA-GFP versus AK-SMAA-GFP-hAR mice treated with/without the ARIs AL1576 Bars represent the mean ± S.E.M of a minimum of 3 scans. * p<0.05. The gel illustrates the representative appearance of one analysis.

In addition to VEGF, the development of diabetic retinopathy has been linked to the deregulation of other growth factors such as TGF-β, IGF-1, and bFGF [37], [42]. Compared to 18 week AK-SMAA-GFP mice, the retinal expression of TGF-β, IGF-1, and bFGF increased in the neural retinas of AK-SMAA-GFP-hAR mice and the expression of these growth factors was reduced in similar AK-SMAA-GFP-hAR mice treated with AL1576 (Fig. 6A, C, E).

Changes in cellular signaling can also accompany these growth factor expression changes. To investigate this possibility, expression levels of the pathway signals P-Akt, P-SAPK/JNK, and P-Erk1/2 (P-44/42 MAPK) from the neural retinas from 4-month old AK-SMAA-GFP mice were compared to those from AK-SMAA-GFP-hAR mice treated with/without the ARI. Compared to AK-SMAA-GFP mice increased signaling in P-Akt, P-SAPK/JNK, and P-ERK1/2 were observed in the Akita-SMAA-GFP-hAR mice and these were reduced by the administration of ARI (Fig. 6B, D, F).

**Figure 7:**
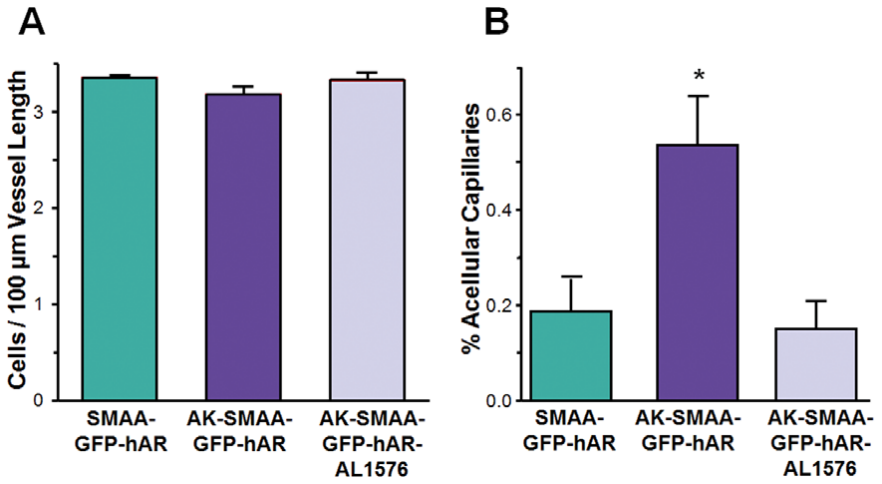
Changes in isolated retinal capillary capillaries from 18 week diabetic AK-SMAA-GFP mice and AK-SMAA-GFP-hAR mice treated with/without ARI. In **A** the capillary cell density expressed as capillary nuclei/100 µm of capillary length is presented. In **B** the percent of acellular capillaries present in the examined neural retinal capillaries is presented. n = 5–7; mean ± S.E.M. * p≤0.03.

To determine if the observed changes in growth factors and signaling are also associated with retinal vascular changes, histological studies were conducted on intact retinal capillaries isolated from a minimum of five 18 week diabetic AK-SMAA-GFP-hAR and AK-SMAA-GFP-hAR mice treated with AL1576 along with age-matched nondiabetic SMAA-GFP-hAR mice. For these studies, the PAS-hematoxylin stained capillary beds in the central areas of each of 4 equal quadrants located mid-distance between the optic nerve and outer retinal edge of each isolated intact retinal vasculature were examined by light microscopy. This represents approximately 1.1 mm^2^ of the central areas of each mouse retina being analyzed. In these stained isolated preparations, the appearance of nuclei of capillary pericyte versus endothelial cells were difficult to differentiate with the classical appearance of pericyte ghosts where basement membrane outlining a cell with no stained nucleus not evident. Therefore, the total number of nuclei present in each capillary was counted as well as the length of each counted capillary. The results were then expressed as capillary nuclei/100 µm of capillary length. Using this approach, a small decrease in the number of nuclei/capillary length was observed in the AK-SMAA-GFP-hAR mice compared to either the AK-SMAA-GFP-hAR mice treated with AL1576 or AK-SMAA-GFP mice (Fig. 7A). The lengths of acellular capillaries present in the analyzed areas were also compared to the total lengths of cellular capillaries present and this was expressed as the percent acellular capillaries present. A comparison of all three mouse groups revealed a small, but significant increase in the percent acellular vessels present in the diabetic AK-SMAA-GFP-hAR mice compared to either AK-SMAA-GFP-hAR mice (p = 0.007) or AK-SMAA-GFP (p = 0.03) mice (Fig. 7B). This data indicates a link between increased aldose reductase expression and retinal capillary cell death that is also consistent with the changes in growth factors and signaling observed.

## Discussion

The ability to modify specific biochemical parameters through gene manipulation make mice a versatile animal model for investigating the biochemical mechanisms of DR. Since experimentally inducing diabetes in mice with chemical agents requires protracted administration of multiple “sub-diabetogenic” doses rather than a single high dose because of increased mortality [24], [43], the time of onset and severity of DM can be inconsistent. This potential problem was circumvented by introducing DM into these transgenic mice by cross-breeding with non-obese spontaneously diabetic C57BL/6-Ins^Akita^ mice which possess a mutation in the insulin 2 gene that causes misfolding of the insulin protein [36]. Akita mice demonstrate loss of beta-cell function, decreased pancreatic beta-cell density, and significant hyperglycemia, as early as 4 weeks of age. In the present study novel transgenic mice with/without DM expressing either GFP alone or GFP and hAR in all vascular tissues expressing smooth muscle alpha have been established. GFP was introduced to aid in the differentiation of vascular pericytes and endothelial cells by fluorescent confocal microscopy while hAR was introduced to increase the relative levels of AR in retinal pericytes.

Compared to AK-SMAA-GFP mice, retinal levels of all four growth factors examined (VEGF, bFGF, IGF-1, and TGF-β) increased in the AK-SMAA-GFP-hAR mice (Fig. 4 and Fig. 6). These growth factors play a central role in regulating the development and progression of DR [44], [45]. Their induction is linked to the development of vascular hyper permeability and altered blood flow which lead to the development of retinal ischemia/hyper permeability that result in capillary wall thickening, pericyte degeneration, endothelial cell loss, and leucocyte adhesion [37]. VEGF, bFGF, IGF-1, and TGF-β are assumed to be secreted by the ischemic retina in order to stimulate residual vessels to proliferate. Of these angiogenic growth factors, VEGF is the most targeted retinal growth factor in the retina. It is upregulated in rat vessels soon after the induction of DM and has been linked to increased vascular permeability in the early stages of DR development [44]. In the later stages of DR, it induces endothelial cell proliferation and chemotaxis. VEGF has also been reported to have a nonvascular role in neuroprotection; however, increased levels of VEGF in the diabetic retina do not appear to prevent neurodegeneration of either retinal ganglion cells or RPE [46].

The first growth factor to be identified in the retina is bFGF and it has been observed to act synergistically with hypoxia to both induce mitogenesis and upregulate VEGF in smooth muscle and endothelial cells [47], [48]. While *in vitro* studies show that endothelial cells secrete bFGF, contact between pericytes and endothelial cells also induces the secretion of TGF-β to presumably counteract the mitogenic effects of bFGF in endothelial cells [49]. TGF- β, which is also secreted by the RPE, has been reported to decrease the growth of bovine aortic endothelial cells [38]. That is why it has been proposed that endothelial cell growth may be controlled by an active equilibrium between TGF- β and bFGF. The first growth factor to be directly linked to DR is IGF-1. Its angiogenic effects and role in neovascularization are documented and IGF-1 levels increase in animal models prior to proliferative DR [44]. IGF also has a neuroprotective effect on retinal ganglion cells and amacrine cells [50]. While bFGF, IGF-1, and VEGF all have similar angiographic effects, VEGF but not IGF-1 or bFGF, is mainly responsible for changes in cellular permeability observed in retinal endothelial cells.

The present studies implicate increased sorbitol levels that are linked to increased levels of hAR rather than insulin deficiency to the induction of these growth factors. This is because diabetic AK-SMAA-GFP mice not expressing hAR were used as the control and growth factor induction was reduced in the diabetic AK-SMAA-GFP-hAR mice treated with ARI (Fig. 4). Since AR and sorbitol accumulation have been linked to pericyte degeneration [10], [14], [23], the induction of VEGF, bFGF, and IGF-1, may be a protective response of the retina to changes in retinal blood flow or potential hypoxia. Similarly, the induction of TGF-β may be a protective response to the increased retinal presence of bFGF.

Growth factor induction has been documented to result in cellular signaling changes in Akt, Erk1/2 (44/42 MAPK) and JNK (SAPK/JNK). Activation (phosphorylation) of Akt is involved in cell survival while JNK and Erk1/2 are stress-activated kinases that can eventually lead to apoptosis. For example, MAPKs are activated by ischemia [51], peroxide induced RPE cell death [52], and hyperglycemia-induced pro-inflammatory responses by retinal Müller glia that is regulated by the receptor for advanced glycation end-products (RAGE) [53]. In the present studies induction of signaling was observed in AK-SMAA-GFR-hAR mice and their induction was reduced by ARI treatment. Since similar induction of growth factors was observed, the observed signaling changes may be secondary to the growth factor induction.

Initial vascular changes were also observed in the isolated retinal vasculature from 18 week diabetic AK-SMAA-GFP-hAR mice where hAR expression had been increased compared to AK-SMAA-GFP mice. These changes included an apparent decrease in capillary cell density (nuclei per capillary length) and an increase in the presence of acellular capillaries which represent areas of nonperfusion. These changes were not observed in AK-SMAA-GFP-hAR mice treated with AL1576 suggesting that these vascular changes were linked to AR activity.

Previous studies in galactose-fed dogs have demonstrated that retinal changes that mirror both histological and clinical changes associated with all stages of DR are initiated by the AR linked degeneration of retinal capillary pericytes [8], [10], [14]. Studies in diabetic and galactose-fed rats also support a central role for AR in DR [54], [55]. However, vascular changes in mice are conflicting and retinal levels of AR are not well-established. Changes in isolated retinal vessels range from a 16% loss of pericytes in 26-week spontaneous diabetic XLacZ mice with no mention of acellular capillary formation [56], to a modest increase in acellular capillary formation (increase from 8 (control) to 11 (diabetic) per square millimeter) and no identified loss in pericytes in 31–34 week diabetic Akita mice [57]. In streptozotocin induced diabetic BLAB/c mice, only the presence of basement membrane thickening and no report on acellular capillary formation or pericyte loss has been reported after 72 weeks [58]. Studies with AR are also conflicting with results ranging from reports that signs of DR that include blood-retinal barrier breakdown, loss of pericytes, neuro-retinal apoptosis, glial reactivation, and the proliferation of blood vessels, are absent in 60 week diabetic mice where AR is knocked-out (AR(−/−) db/db) [18], to reports that mice with short-term streptozotocin-induced diabetes lack many of the retinal biochemical changes such as lower VEGF protein expression, increased rather than reduced enzyme oxidative defense systems (with the exception of catalase), and lower retinal sorbitol levels compared to diabetic rats [27]. By expressing hAR in their vascular retinal pericytes, AK-SMAA-GFP-hAR mice demonstrate vascular changes after 18 of diabetes, a time frame much earlier that the 31–34 week period for diabetic Akita mice [57]. The vascular changes in AK-SMAA-GFP-hAR mice are anticipated to increase with longer durations of DM; however, additional long-term studies are required to confirm this premise.

Since neurodegeneration in diabetic animal models can potentially be detected by ERG [59], preliminary ERG studies were conducted to determine whether neurodegeneration occurs in the AK-SMAA-GFP-hAR mice. Compared to nondiabetic SMAA-GFP-hAR mice, AK-SMAA-GFP-hAR mice demonstrated a decrease in the b-wave maximum response (Fig. 5). This decrease was not observed in similar diabetic mice treated with AL1576. ERG studies in SMAA-GFP mice have previously established that expression of GFP does not adversely affect retinal function [43], [60]. Therefore, we can conclude from these preliminary studies that the ERG changes are associated with AR activity and sorbitol accumulation. Similar inhibition of AR in diabetic rats with either fiderestat (SNK-860) or TAT has also been reported to ameliorate ERG changes in b-wave oscillatory potentials [61], [62].

Since the same smooth muscle alpha actin promoter was used to introduce GFP and hAR into these transgenic mice, it is assumed that the tissue distribution of GFP and hAR is similar. Based on our studies on the importance of pericyte degeneration on the progression of DR in dogs, we focused on the induction of hAR into the retinal capillary cells. Using confocal microscopy, we have confirmed the select presence of GFP in the retinal capillary pericytes of mice in the present study (Fig. 1) while others have also reported the presence of GFP in pericytes of the initial SMAA-GFP used to establish our colony [31], [32]. However, retinal cone bipolar cells [33], the rod inner segments [63], Müller cells [64] and retinal pigment epithelia (RPE) [65] also contain smooth muscle alpha actin. Immunohistochemical studies also indicate that AR, in addition to being specifically localized in retinal capillary pericytes, is present in Mueller cells, some ganglion and cone cells, and in the axons in the optic nerve of human eyes [66], [67]. Similarly, in db/db mice, AR is present in Mueller cells, and neuronal cells in both the retinal ganglion cell and the inner nuclear layer (INL) as well as in capillary pericytes [18]. AR is also present in retinal pigmented epithelial (RPE) cells [68], [69].

Because the neural retinas were mechanical separated from the poster segment leaving the retinal pigmented epithelium and choroid still attached to the posterior globe, the present results are limited to changes in the neural retina. The retinal vasculature represents only a small component of the entire neural retina and our previous studies in dog suggest that the vascular contribution to neural retinal sorbitol levels is less than 10% of the total sorbitol measured. Therefore, the 30% increase in retinal sorbitol observed in Fig. 3 includes the contribution by other retinal cells containing smooth muscle alpha actin. Although lens sorbitol levels in the mouse lens are extremely low, the slight increase in lens sorbitol levels observed for AK-SMAA-GFP-hAR mice support this premise since the lens also contains smooth muscle alpha actin. Therefore, future immunohistochemical studies of AR localization are planned in order to clarify the distribution and induction of AR in these transgenic mice.

In summary, the present studies suggest that these novel diabetic mice expressing GFP and hAR in vascular tissues possessing smooth muscle alpha actin serve as new animal models for investigating. The AK-SMAA-GFP-hAR expressing mice show increased retinal sorbitol levels that are linked to growth factor and signaling expression changes similar to those observed in diabetic rats. In preliminary ERG studies, these mice also show AR-linked neurological changes. The importance of AR activity in these retinal changes is verified by the ability of ARIs to ameliorate the AR-associated changes. Therefore, these mice provide a new animal model for investigating the role of AR in diabetic retinopathy. In addition to these biochemical changes, the presence of GFP should be beneficial in the evaluation of retinal vascular changes.

## References

[1] Kuwabara T, Carroll JM, Cogan DG (1961) Retinal vascular patterns. III. Age, hypertension, absolute glaucoma, injury. Arch Ophthalmol 65: 708–716. 13755463 10.1001/archopht.1961.01840020710019

[2] Cogan DG, Toussaint D, Kuwabara T (1961) Retinal vascular patterns. IV. Diabetic retinopathy. Arch Ophthalmol 66: 366–378. 13694291 10.1001/archopht.1961.00960010368014

[3] Betsholtz C, Lindblom P, Gerhardt H (2005) Role of pericytes in vascular morphogenesis. Exs 115–125. 15617474 10.1007/3-7643-7311-3_8

[4] Chan LS, Li WY, Khatami M, Rockey JH (1986) Actin in cultured bovine retinal capillary pericytes: morphological and functional correlation. Exp Eye Res 43: 41–54. 2942415 10.1016/s0014-4835(86)80044-6

[5] Antonelli-Orlidge A, Smith SR, D'Amore PA (1989) Influence of pericytes on capillary endothelial cell growth. Am Rev Respir Dis 140: 1129–1131. 2679268 10.1164/ajrccm/140.4.1129

[6] Kador PF, Akagi Y, Takahashi Y, Ikebe H, Wyman M, et al (1990) Prevention of retinal vessel changes associated with diabetic retinopathy in galactose-fed dogs by aldose reductase inhibitors. Arch Ophthalmol 108: 1301–1309. 2119169 10.1001/archopht.1990.01070110117035

[7] Kador PF, Akagi Y, Terubayashi H, Wyman M, Kinoshita JH (1988) Prevention of pericyte ghost formation in retinal capillaries of galactose-fed dogs by aldose reductase inhibitors. Arch Ophthalmol 106: 1099–1102. 3401138 10.1001/archopht.1988.01060140255036

[8] Kador PF, Takahashi Y, Akagi Y, Neuenschwander H, Greentree W, et al (2002) Effect of galactose diet removal on the progression of retinal vessel changes in galactose-fed dogs. Invest Ophthalmol Vis Sci 43: 1916–1921. 12036999

[9] Kador PF, Takahashi Y, Wyman M, Ferris F 3rd (1995) Diabeteslike proliferative retinal changes in galactose-fed dogs. Arch Ophthalmol 113: 352–354. 7887849 10.1001/archopht.1995.01100030108031

[10] Neuenschwander H, Takahashi Y, Kador PF (1997) Dose-dependent reduction of retinal vessel changes associated with diabetic retinopathy in galactose-fed dogs by the aldose reductase inhibitor M79175. J Ocul Pharmacol Ther 13: 517–528. 9436155 10.1089/jop.1997.13.517

[11] Takahashi Y, Augustin W, Wyman M, Kador PF (1993) Quantitative analysis of retinal vessel changes in galactose-fed dogs. J Ocul Pharmacol 9: 257–269. 8228533 10.1089/jop.1993.9.257

[12] Takahashi Y, Wyman M, Ferris F 3rd, Kador PF (1992) Diabeteslike preproliferative retinal changes in galactose-fed dogs. Arch Ophthalmol 110: 1295–1302. 1520120 10.1001/archopht.1992.01080210113037

[13] Akagi Y, Terubayashi H, Millen J, Kador PF, Kinoshita JH (1986) Aldose reductase localization in dog retinal mural cells. Curr Eye Res 5: 883–886. 3096638 10.3109/02713688609029241

[14] Murata M, Ohta N, Fujisawa S, Tsai JY, Sato S, et al (2002) Selective pericyte degeneration in the retinal capillaries of galactose-fed dogs results from apoptosis linked to aldose reductase-catalyzed galactitol accumulation. J Diabetes Complications 16: 363–370. 12200082 10.1016/s1056-8727(01)00171-4

[15] Sato S, Secchi EF, Lizak MJ, Fukase S, Ohta N, et al (1999) Polyol formation and NADPH-dependent reductases in dog retinal capillary pericytes and endothelial cells. Invest Ophthalmol Vis Sci 40: 697–704. 10067973

[16] Robison WG (2000) Galactosemic Animal Models; Sima AAF, Shafir E, editors. Amsterdam: Harwood Academic Publishers. 273–308 p.

[17] Yamaoka T, Nishimura C, Yamashita K, Itakura M, Yamada T, et al (1995) Acute onset of diabetic pathological changes in transgenic mice with human aldose reductase cDNA. Diabetologia 38: 255–261. 7758869 10.1007/BF00400627

[18] Cheung AK, Fung MK, Lo AC, Lam TT, So KF, et al (2005) Aldose Reductase Deficiency Prevents Diabetes-Induced Blood-Retinal Barrier Breakdown, Apoptosis, and Glial Reactivation in the Retina of db/db Mice. Diabetes 54: 3119–3125. 16249434 10.2337/diabetes.54.11.3119

[19] Frank RN, Keirn RJ, Kennedy A, Frank KW (1983) Galactose-induced retinal capillary basement membrane thickening: prevention by Sorbinil. Invest Ophthalmol Vis Sci 24: 1519–1524. 6642931

[20] Robison WG Jr, Kador PF, Kinoshita JH (1983) Retinal capillaries: basement membrane thickening by galactosemia prevented with aldose reductase inhibitor. Science 221: 1177–1179. 6612330 10.1126/science.6612330

[21] Robison WG Jr, McCaleb ML, Feld LG, Michaelis OEt, Laver N, et al (1991) Degenerated intramural pericytes (‘ghost cells’) in the retinal capillaries of diabetic rats. Curr Eye Res 10: 339–350. 1829996 10.3109/02713689108996340

[22] Tilton RG, LaRose LS, Kilo C, Williamson JR (1986) Absence of degenerative changes in retinal and uveal capillary pericytes in diabetic rats. Invest Ophthalmol Vis Sci 27: 716–721. 3700020

[23] Kador PF, Randazzo J, Blessing K, Makita J, Zhang P, et al (2009) Polyol formation in cell lines of rat retinal capillary pericytes and endothelial cells (TR-rPCT and TR-iBRB). J Ocul Pharmacol Ther 25: 299–308. 19450153 10.1089/jop.2008.0070 PMC2958435

[24] Wilson GL, Leiter EH (1990) Streptozotocin interactions with pancreatic beta cells and the induction of insulin-dependent diabetes. Curr Top Microbiol Immunol 156: 27–54. 2143132 10.1007/978-3-642-75239-1_3

[25] Tilton RG, Miller EJ, Kilo C, Williamson JR (1985) Pericyte form and distribution in rat retinal and uveal capillaries. Invest Ophthalmol Vis Sci 26: 68–73. 3967956

[26] Cuthbertson RA, Mandel TE (1986) Anatomy of the mouse retina. Endothelial cell-pericyte ratio and capillary distribution. Invest Ophthalmol Vis Sci 27: 1659–1664. 3771146

[27] Obrosova IG, Drel VR, Kumagai AK, Szabo C, Pacher P, et al (2006) Early diabetes-induced biochemical changes in the retina: comparison of rat and mouse models. Diabetologia 49: 2525–2533. 16896942 10.1007/s00125-006-0356-7 PMC2228251

[28] Wang J, Niu W, Nikiforov Y, Naito S, Chernausek S, et al (1997) Targeted overexpression of IGF-I evokes distinct patterns of organ remodeling in smooth muscle cell tissue beds of transgenic mice. J Clin Invest 100: 1425–1439. 9294108 10.1172/JCI119663 PMC508321

[29] Tsai J, Yamamoto T, Fariss R, Hickman F, Pagan-Mercado G (2002) Using SMAA-GFP Mice to Study Pericyte Coverage of Retinal Vessels. Invest Ophthalmol Vis Sci 43: 192.

[30] Tsai J-Y, Yu Z-X, Wawrousek E (2001) Generation and characterization of SMAA-GFP mice. Invest Opthalmol Vis Sci 42: 2001.

[31] Seeliger MW, Beck SC, Pereyra-Munoz N, Dangel S, Tsai JY, et al (2005) In vivo confocal imaging of the retina in animal models using scanning laser ophthalmoscopy. Vision Res 45: 3512–3519. 16188288 10.1016/j.visres.2005.08.014

[32] Yokota T, Kawakami Y, Nagai Y, Ma JX, Tsai JY, et al (2006) Bone marrow lacks a transplantable progenitor for smooth muscle type alpha-actin-expressing cells. Stem Cells 24: 13–22. 16099999 10.1634/stemcells.2004-0346

[33] Hickman F, Fariss R, Pagan-Mercado, Yamamoto T, Wawrousek E, et al (2002) Characterization Of Retinal Neurons Over-expressing. Human Aldose Reductase In Smaa-har Mice Invest Opthalmol Vis Sci 43: 742.

[34] Bradford MM (1976) A rapid and sensitive method for the quantitation of microgram quantities of protein utilizing the principle of protein-dye binding. Anal Biochem 72: 248–254. 942051 10.1016/0003-2697(76)90527-3

[35] Nystuen AM, Sachs AJ, Yuan Y, Heuermann L, Haider NB (2008) A novel mutation in Prph2, a gene regulated by Nr2e3, causes retinal degeneration and outer-segment defects similar to Nr2e3 (rd7/rd7) retinas. Mamm Genome 19: 623–633. 18763016 10.1007/s00335-008-9138-5 PMC4910819

[36] Yoshioka M, Kayo T, Ikeda T, Koizumi A (1997) A novel locus, Mody4, distal to D7Mit189 on chromosome 7 determines early-onset NIDDM in nonobese C57BL/6 (Akita) mutant mice. Diabetes 46: 887–894. 9133560 10.2337/diab.46.5.887

[37] Mochizuki M, Sugita S, Ishikawa N, Watanabe T (2000) Immunoregulation by aqueous humor. Cornea 19: S24–25. 10832718 10.1097/00003226-200005001-00006

[38] Chen Y, Mehta G, Vasiliou V (2009) Antioxidant defenses in the ocular surface. Ocul Surf 7: 176–185. 19948101 10.1016/s1542-0124(12)70185-4 PMC4104792

[39] Obrosova IG, Minchenko AG, Vasupuram R, White L, Abatan OI, et al (2003) Aldose reductase inhibitor fidarestat prevents retinal oxidative stress and vascular endothelial growth factor overexpression in streptozotocin-diabetic rats. Diabetes 52: 864–871. 12606532 10.2337/diabetes.52.3.864

[40] Amano S, Yamagishi S, Kato N, Inagaki Y, Okamoto T, et al (2002) Sorbitol dehydrogenase overexpression potentiates glucose toxicity to cultured retinal pericytes. Biochem Biophys Res Commun 299: 183–188. 12437967 10.1016/s0006-291x(02)02584-6

[41] Frank RN, Amin R, Kennedy A, Hohman TC (1997) An aldose reductase inhibitor and aminoguanidine prevent vascular endothelial growth factor expression in rats with long-term galactosemia. Arch Ophthalmol 115: 1036–1047. 9258227 10.1001/archopht.1997.01100160206011

[42] Van Geest RJ, Klaassen I, Vogels IM, Van Noorden CJ, Schlingemann RO (2010) Differential TGF-{beta} signaling in retinal vascular cells: a role in diabetic retinopathy? Invest Ophthalmol Vis Sci 51: 1857–1865. 19959647 10.1167/iovs.09-4181

[43] Deeds MC, Anderson JM, Armstrong AS, Gastineau DA, Hiddinga HJ, et al (2011) Single dose streptozotocin-induced diabetes: considerations for study design in islet transplantation models. Lab Anim 45: 131–140. 21478271 10.1258/la.2010.010090 PMC3917305

[44] Cai J, Boulton M (2002) The pathogenesis of diabetic retinopathy: old concepts and new questions. Eye 16: 242–260. 12032713 10.1038/sj.eye.6700133

[45] Welge-Lussen U, May CA, Neubauer AS, Priglinger S (2001) Role of tissue growth factors in aqueous humor homeostasis. Curr Opin Ophthalmol 12: 94–99. 11224714 10.1097/00055735-200104000-00003

[46] Iyengar L, Patkunanathan B, Lynch OT, McAvoy JW, Rasko JE, et al (2006) Aqueous humour- and growth factor-induced lens cell proliferation is dependent on MAPK/ERK1/2 and Akt/PI3-K signalling. Exp Eye Res 83: 667–678. 16684521 10.1016/j.exer.2006.03.008

[47] Stavri GT, Zachary IC, Baskerville PA, Martin JF, Erusalimsky JD (1995) Basic fibroblast growth factor upregulates the expression of vascular endothelial growth factor in vascular smooth muscle cells. Synergistic interaction with hypoxia. Circulation 92: 11–14. 7788904 10.1161/01.cir.92.1.11

[48] Seghezzi G, Patel S, Ren CJ, Gualandris A, Pintucci G, et al (1998) Fibroblast growth factor-2 (FGF-2) induces vascular endothelial growth factor (VEGF) expression in the endothelial cells of forming capillaries: an autocrine mechanism contributing to angiogenesis. J Cell Biol 141: 1659–1673. 9647657 10.1083/jcb.141.7.1659 PMC2132998

[49] Antonelli-Orlidge A, Saunders KB, Smith SR, D'Amore PA (1989) An activated form of transforming growth factor beta is produced by cocultures of endothelial cells and pericytes. Proc Natl Acad Sci U S A 86: 4544–4548. 2734305 10.1073/pnas.86.12.4544 PMC287307

[50] Barber AJ, Nakamura M, Wolpert EB, Reiter CE, Seigel GM, et al (2001) Insulin rescues retinal neurons from apoptosis by a phosphatidylinositol 3-kinase/Akt-mediated mechanism that reduces the activation of caspase-3. J Biol Chem 276: 32814–32821. 11443130 10.1074/jbc.M104738200

[51] Dreixler JC, Bratton A, Du E, Shaikh AR, Savoie B, et al (2011) Mitogen-activated protein kinase phosphatase-1 (MKP-1) in retinal ischemic preconditioning. Exp Eye Res 93: 340–349. 21094639 10.1016/j.exer.2010.10.011 PMC3074007

[52] Ho TC, Yang YC, Cheng HC, Wu AC, Chen SL, et al (2006) Activation of mitogen-activated protein kinases is essential for hydrogen peroxide -induced apoptosis in retinal pigment epithelial cells. Apoptosis 11: 1899–1908. 16927023 10.1007/s10495-006-9403-6

[53] Zong H, Ward M, Madden A, Yong PH, Limb GA, et al (2010) Hyperglycaemia-induced pro-inflammatory responses by retinal Muller glia are regulated by the receptor for advanced glycation end-products (RAGE). Diabetologia 53: 2656–2666. 20835858 10.1007/s00125-010-1900-z

[54] Robison WG, Laver NM, Lou MF, editors (1995) The Role of Aldose Reductase in Diabetic Retinopathy: Prevention and Intervention Studies. Oxford: Pergamon. 593–639 p.

[55] Sun W, Oates PJ, Coutcher JB, Gerhardinger C, Lorenzi M (2006) A selective aldose reductase inhibitor of a new structural class prevents or reverses early retinal abnormalities in experimental diabetic retinopathy. Diabetes 55: 2757–2762. 17003340 10.2337/db06-0138

[56] Pfister F, Feng Y, vom Hagen F, Hoffmann S, Molema G, et al (2008) Pericyte migration: a novel mechanism of pericyte loss in experimental diabetic retinopathy. Diabetes 57: 2495–2502. 18559662 10.2337/db08-0325 PMC2518502

[57] Barber AJ, Antonetti DA, Kern TS, Reiter CE, Soans RS, et al (2005) The Ins2Akita mouse as a model of early retinal complications in diabetes. Invest Ophthalmol Vis Sci 46: 2210–2218. 15914643 10.1167/iovs.04-1340

[58] Cuthbertson RA, Mandel TE (1987) The effect of murine fetal islet transplants on renal and retinal capillary basement membrane thickness. Transplant Proc 19: 2919–2921. 3105130

[59] Ozawa Y, Kurihara T, Sasaki M, Ban N, Yuki K, et al (2011) Neural degeneration in the retina of the streptozotocin-induced type 1 diabetes model. Exp Diabetes Res 2011: 108328. 22144984 10.1155/2011/108328 PMC3226536

[60] Beck SC, Schaeferhoff K, Michalakis S, Fischer MD, Huber G, et al (2010) In vivo analysis of cone survival in mice. Invest Ophthalmol Vis Sci 51: 493–497. 19737879 10.1167/iovs.09-4003

[61] Hotta N, Koh N, Sakakibara F, Nakamura J, Hamada Y, et al (1995) An aldose reductase inhibitor, TAT, prevents electroretinographic abnormalities and ADP-induced hyperaggregability in streptozotocin-induced diabetic rats. Eur J Clin Invest 25: 948–954. 8719936 10.1111/j.1365-2362.1995.tb01972.x

[62] Hotta N, Koh N, Sakakibara F, Nakamura J, Hamada Y, et al (1995) Effect of an aldose reductase inhibitor, SNK-860, on deficits in the electroretinogram of diabetic rats. Exp Physiol 80: 981–989. 8962712 10.1113/expphysiol.1995.sp003909

[63] Drenckhahn D, Groschel-Stewart U (1977) Localization of myosin and actin in ocular nonmuscle cells. Immunofluorescence-microscopic, biochemical, and electron-microscopic studies. Cell Tissue Res 181: 493–503. 328160 10.1007/BF00221771

[64] Joussen AM, Doehmen S, Le ML, Koizumi K, Radetzky S, et al (2009) TNF-alpha mediated apoptosis plays an important role in the development of early diabetic retinopathy and long-term histopathological alterations. Mol Vis 15: 1418–1428. 19641635 PMC2716944

[65] Kivela T, Uusitalo M (1998) Structure, development and function of cytoskeletal elements in non-neuronal cells of the human eye. Prog Retin Eye Res 17: 385–428. 9695798 10.1016/s1350-9462(98)00001-9

[66] Akagi Y, Kador PF, Kuwabara T, Kinoshita JH (1983) Aldose reductase localization in human retinal mural cells. Invest Ophthalmol Vis Sci 24: 1516–1519. 6417042

[67] Akagi Y, Yajima Y, Kador PF, Kuwabara T, Kinoshita JH (1984) Localization of aldose reductase in the human eye. Diabetes 33: 562–566. 6427040 10.2337/diab.33.6.562

[68] Vinores SA, Van Niel E, Swerdloff JL, Campochiaro PA (1993) Electron microscopic immunocytochemical evidence for the mechanism of blood-retinal barrier breakdown in galactosemic rats and its association with aldose reductase expression and inhibition. Exp Eye Res 57: 723–735. 8150024 10.1006/exer.1993.1180

[69] Henry DN, Frank RN, Hootman SR, Rood SE, Heilig CW, et al (2000) Glucose-specific regulation of aldose reductase in human retinal pigment epithelial cells in vitro. Invest Ophthalmol Vis Sci 41: 1554–1560. 10798676

